# Blue®m gel vs hyaluronic acid gel in wound healing and pain control following functional crown lengthening: a randomized controlled trial

**DOI:** 10.1007/s44445-026-00178-4

**Published:** 2026-05-12

**Authors:** Riddhi Vyas, Santhosh Kumar, C M Manasa

**Affiliations:** 1 Department of Periodontology, Manipal College of Dental Sciences, Manipal Academy of Higher Education, Manipal, Karnataka India; 2 Department of Pharmaceutics, Manipal College of Pharmaceutical Sciences, Manipal Academy of Higher Education, Manipal, Karnataka India

**Keywords:** Hyaluronic acid, Crown lengthening, Periodontal dressing, Wound healing, Postoperative pain

## Abstract

Periodontal wound dressings have advanced from passive protectants to bioactive agents. This study compared the efficacy of Blue®m gel and a hyaluronic acid (HA) gel in promoting wound healing and controlling postoperative pain after functional crown lengthening surgery. A prospective, randomized, patient and outcome assessor-blinded study was conducted on 40 systemically healthy patients (aged 20–60 years) requiring crown lengthening of posterior teeth. Participants were divided into two groups: Blue®m gel (Group A) and custom-formulated HA gel (Group B). Both gels were applied to the surgical site immediately after suturing and then three times daily for 1 week. Wound healing was assessed at 1, 2, and 3 weeks using the Early Wound Healing Index (EHS, including CSR for closure, CSH for hemostasis and CSI for inflammation) and the Landry Wound Healing Index. Postoperative pain was evaluated via a Numeric Pain Rating Scale (NPRS) on Days 1, 2, and 3. Outcome comparisons between groups were performed with Chi-square tests for categorical healing indices and Mann–Whitney test for ordinal data. Both interventions yielded excellent clinical outcomes with comparable healing patterns. By Week 1, 100% of sites in both groups had complete incision closure (merged margins). Approximately 20% of patients in each group showed slight fibrin presence and mild redness at Week 1, which resolved entirely by Week 2. Consequently, EHS scores were high in both groups (most cases scoring 9–10 at Week 1 and 10 by Week 2) with no significant intergroup difference (*p* > 0.05). The Landry index reflected progressive improvement from predominantly red tissue at Week 1 to fully pink, healthy tissue by Week 3 in both groups (*p* > 0.05). Postoperative pain was mild and diminished rapidly: On Day 1, pain scores ranged from 0 to 4 in Group A and 1 to 2 in Group B, dropping significantly by Day 3 with no difference between the two groups (*p* > 0.05). No adverse effects were observed with either gel. Blue®m gel and HA gel demonstrated favorable clinical outcomes in soft tissue wound healing and early postoperative pain control, with no statistically significant differences observed between groups. Both agents can be safely recommended as effective periodontal dressings in this context, with the choice between them potentially guided by practical considerations such as cost and availability.

**Supplementary Information:**

The online version contains supplementary material available at 10.1007/s44445-026-00178-4.

## Introduction

The evolution of periodontal dressings mirrors advances in dental materials science and a growing emphasis on patient-centered outcomes. Traditional eugenol-based dressings, such as Wonderpak, were eventually replaced by non-eugenol formulations, like Coe-Pack, which offered superior biocompatibility but limited bioactivity (Kathariya et al. 2015). To overcome the passive nature of mechanical dressings, bioactive materials like Hyaluronic Acid (HA) and Blue®m gel have emerged. HA, a glycosaminoglycan present in the extracellular matrix, plays a pivotal role in angiogenesis, inflammation control, and tissue hydration (Necas et al. 2008). Clinical studies have demonstrated its potential in enhancing periodontal wound healing, reducing inflammation, and modulating pain (Eick et al. 2013; Mancini and Mancini 2024). Blue®m gel, developed in the Netherlands, utilizes oxygen-releasing compounds such as sodium perborate and honey enzymes to stimulate tissue regeneration and suppress microbial activity. The gel’s low-concentration hydrogen peroxide release mimics physiological wound oxygenation, fostering neovascularization and fibroblast activation (Juliana and Tarek 2022; Kaur et al. 2024). While both agents have shown individual success in clinical contexts, direct comparative trials are scarce. This study aimed to evaluate and compare their wound healing potential and analgesic efficacy following surgical crown lengthening.

## Materials and methods

### Study design and ethical approval

The presented study was a single-centre, randomized, parallel-arm, patient and outcome assessor blinded clinical trial conducted from July 2024 to January 2025 at the Department of Periodontology, Manipal College of Dental Sciences, Manipal. Ethical approval was obtained from Kasturba Medical College, Kasturba Hospital Ethics committee (IEC No. 92/2023), and the trial was registered with the Clinical Trials Registry of India (CTRI/2024/02/062326). All the patients participating in this clinical trial agreed to sign the informed consent.

### Participants and sample size

Forty systemically healthy participants (aged 20–60 years), each requiring functional crown lengthening involving two or more posterior teeth, were enrolled. The primary outcome variable for sample size estimation was early wound healing, assessed using the Early Wound Healing Index (EHS) at 1 week. Sample size was calculated using G*Power (v3.1) assuming a moderate effect size (d = 0.65) based on previous clinical studies on hyaluronic acid and oxygen-releasing agents (Yıldırım et al. 2018). With α = 0.05 and 80% power, a minimum of 18 subjects per group was required; this was increased to 20 per group to account for possible attrition.

### Inclusion and exclusion criteria

The patients included in the study were adults 20–60 years of age with tooth/teeth indicated for functional crown lengthening for prosthetic/restorative reasons.

Patients who had used antibiotics recently within 6 months, with systemic conditions affecting healing (e.g., diabetes, immunosuppression), smokers, pregnant or lactating women, and patients who had undergone Periodontal surgery in the past 6 months.

### Randomization and blinding

**Fig. 1:**
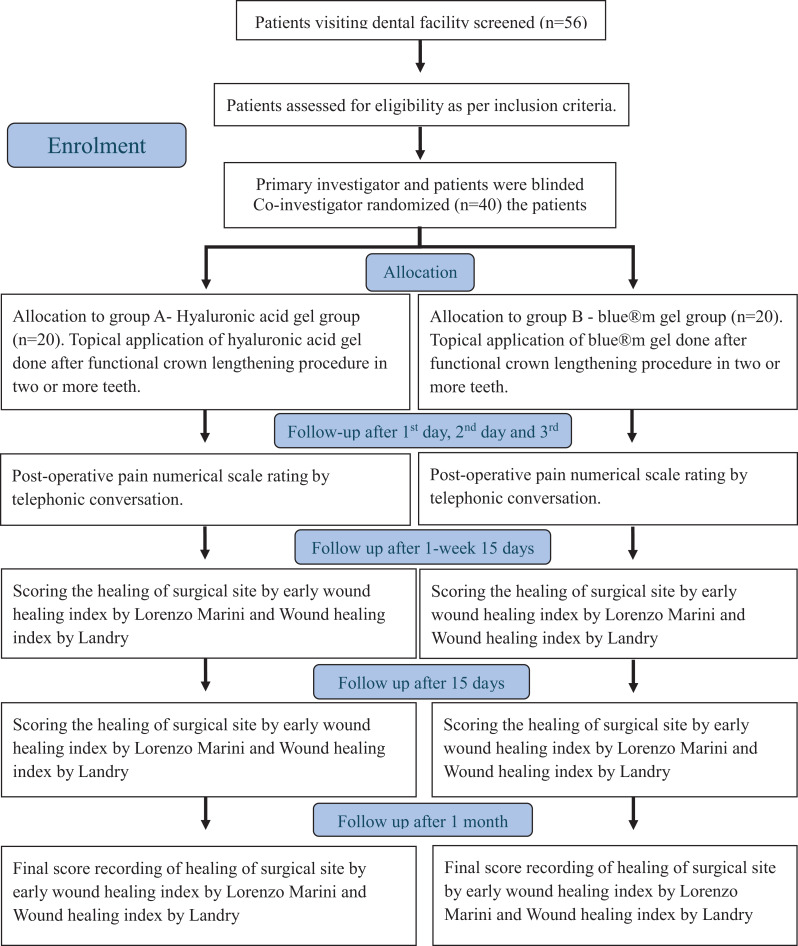
Consort flowchart

Patients were randomly allocated using block randomization. The study gels were dispensed in identical, unlabelled tubes to ensure patient blinding. The operator performed all surgical procedures. Clinical healing assessments were performed using standardized indices. Although the operator conducted postoperative follow-up calls for pain assessment, allocation concealment was maintained, and patients remained blinded to group assignment (Fig. 1).

### Surgical protocol

**Fig. 2:**
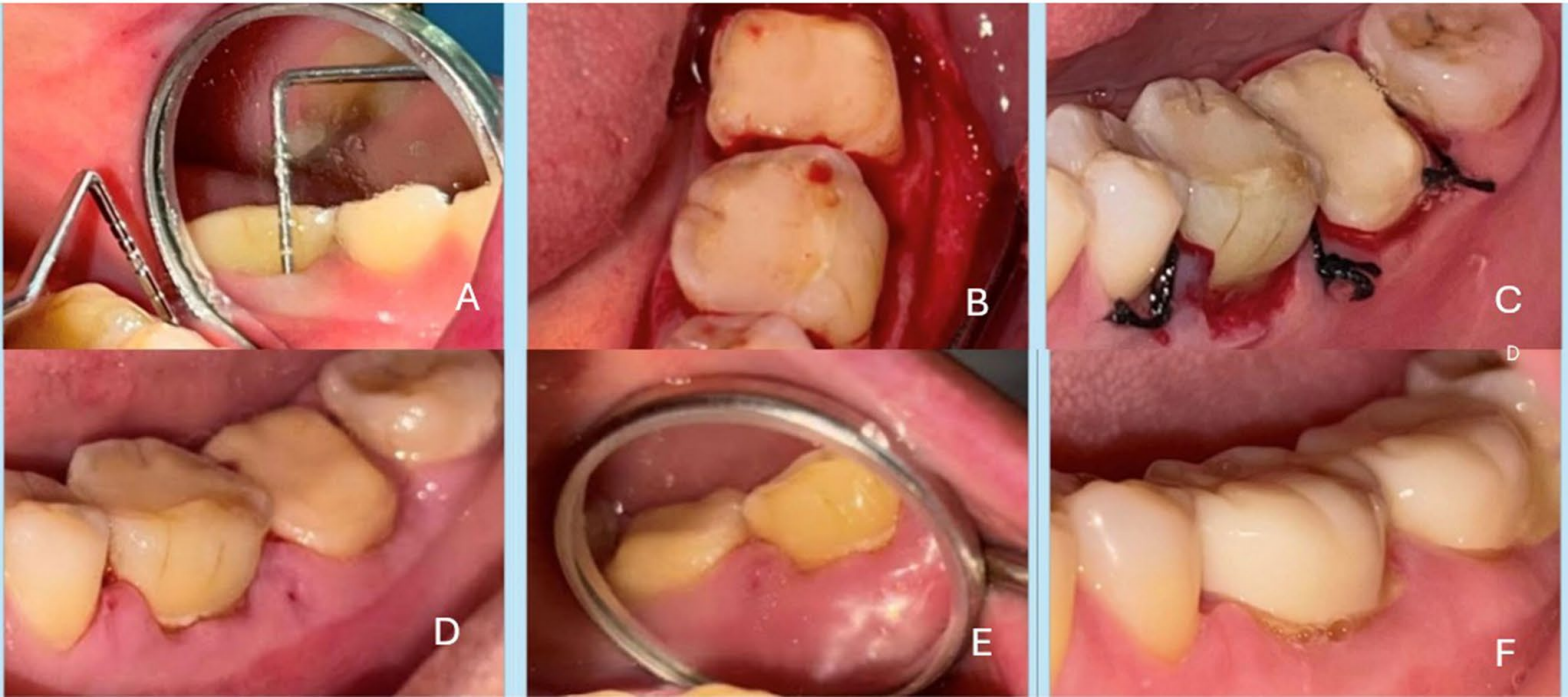
Control group A. (**A**) Pre-op picture before the surgical crown lengthening procedure (**B**) Reflection of full thickness periosteal flap for osseous reduction (**C**) Sutures placed to reapproximate the flap (**D**) Healing after one week of surgery and gel application for control group (**E**) Healing after two weeks of surgery and gel application for control group (**F**) Healing after three weeks of surgery and gel application for control group

**Fig. 3:**
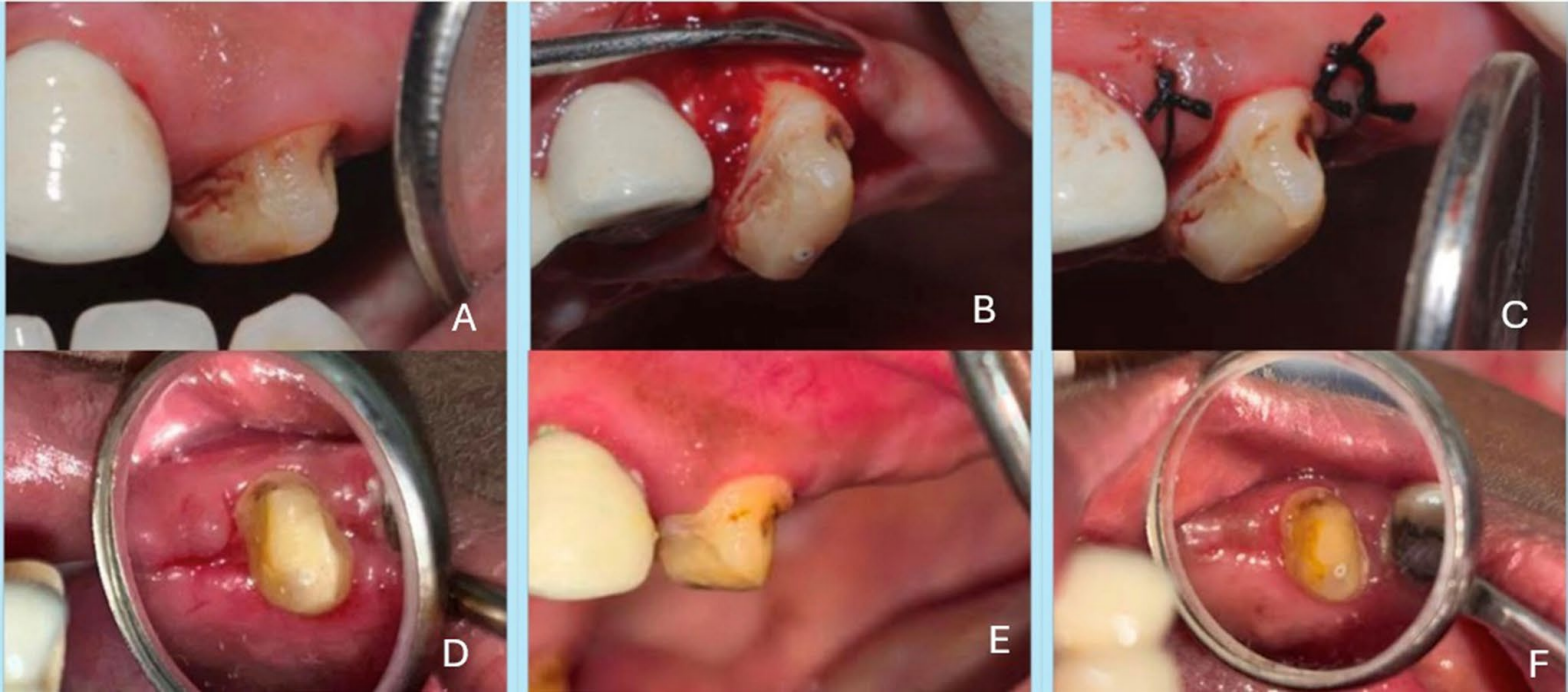
Test group B. (**A**) Pre-op picture before the surgical crown lengthening procedure (**B**) Reflection of full thickness periosteal flap for osseous reduction (**C**) Sutures placed to reapproximate the flap (**D**) Healing after one week of surgery and gel application for test group (**E**) Healing after two weeks of surgery and gel application for test group (**F**) Healing after three weeks of surgery and gel application for test group

All subjects underwent a standardized functional crown lengthening procedure. After adequate local anaesthesia (2% lignocaine with 1:80,000 epinephrine), a full-thickness mucoperiosteal flap was reflected with an internal bevel incision. Granulation tissue was removed, and the underlying bone was recontoured as needed (osteoplasty/ostectomy) to achieve the desired crown length. The flap was then repositioned and sutured with 4–0 black silk sutures using an interrupted suturing technique. Figure 2 (A to F) (Control Group) and Fig. 3 (A to F) (Test Group) illustrates the surgical steps of crown lengthening, from flap reflection to suturing. Post-operatively, patients were not given any systemic or topical analgesics or antibiotics, but instructions were given to all the patients to contact the operator if pain was present.

### Application of study gels

**Fig. 4:**
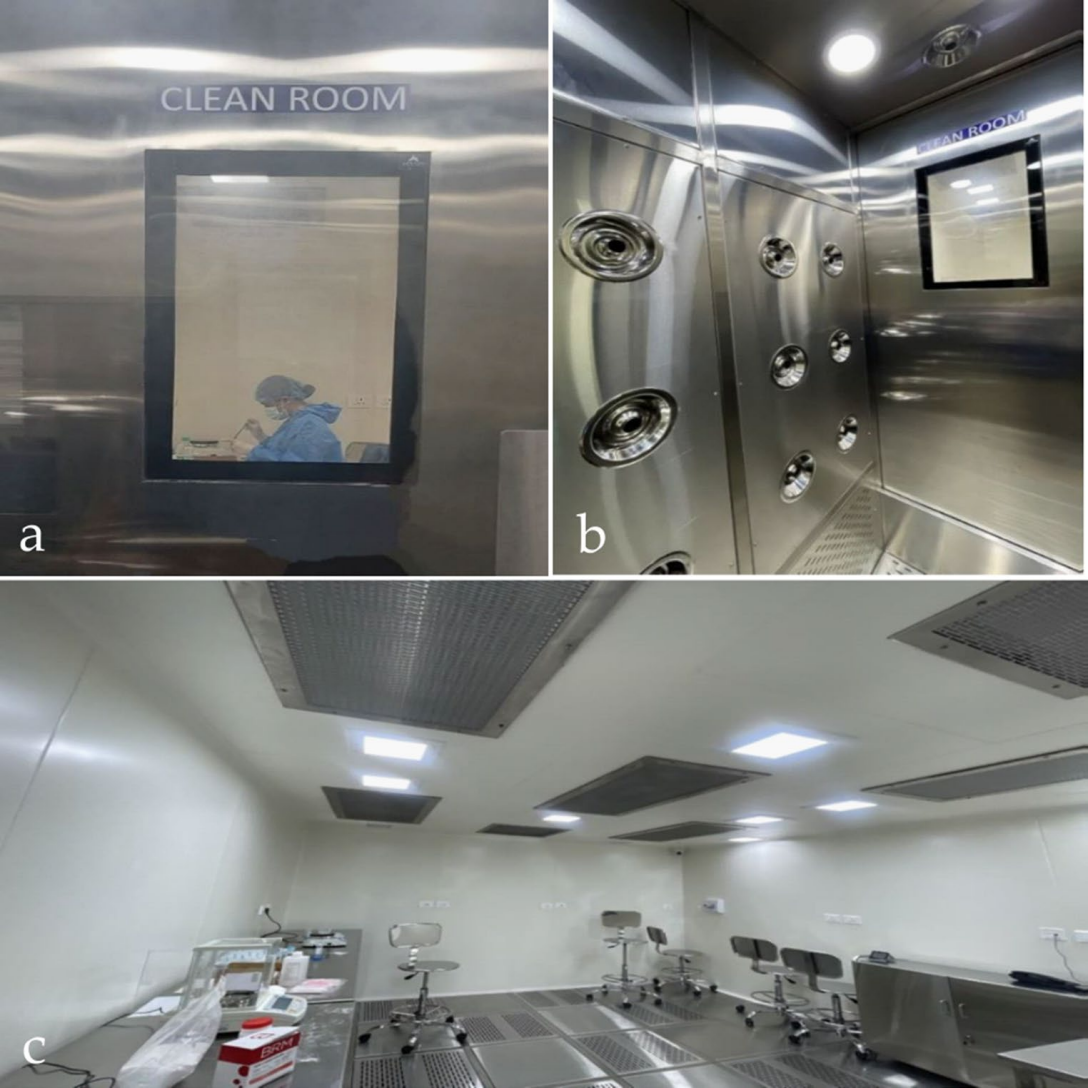
Clean room facility used for hyaluronic acid gel preparation (**a**) photo of ‘clean room’ from outside the facility door (**b**) entrance for donning and decontamination (**c**) inside the facility

Group A (Blue®m gel): This group received a commercially available oxygen-releasing gel containing sodium perborate, honey-derived enzymes, and lactoferrin. The Blue®m gel was used as provided by the manufacturer without further modification. It was divided into separate containers in a clean-room facility (ISO class 4) (Fig. 4) to maintain sterility, and then they were coded to reduce bias during allocation.

**Fig. 5:**
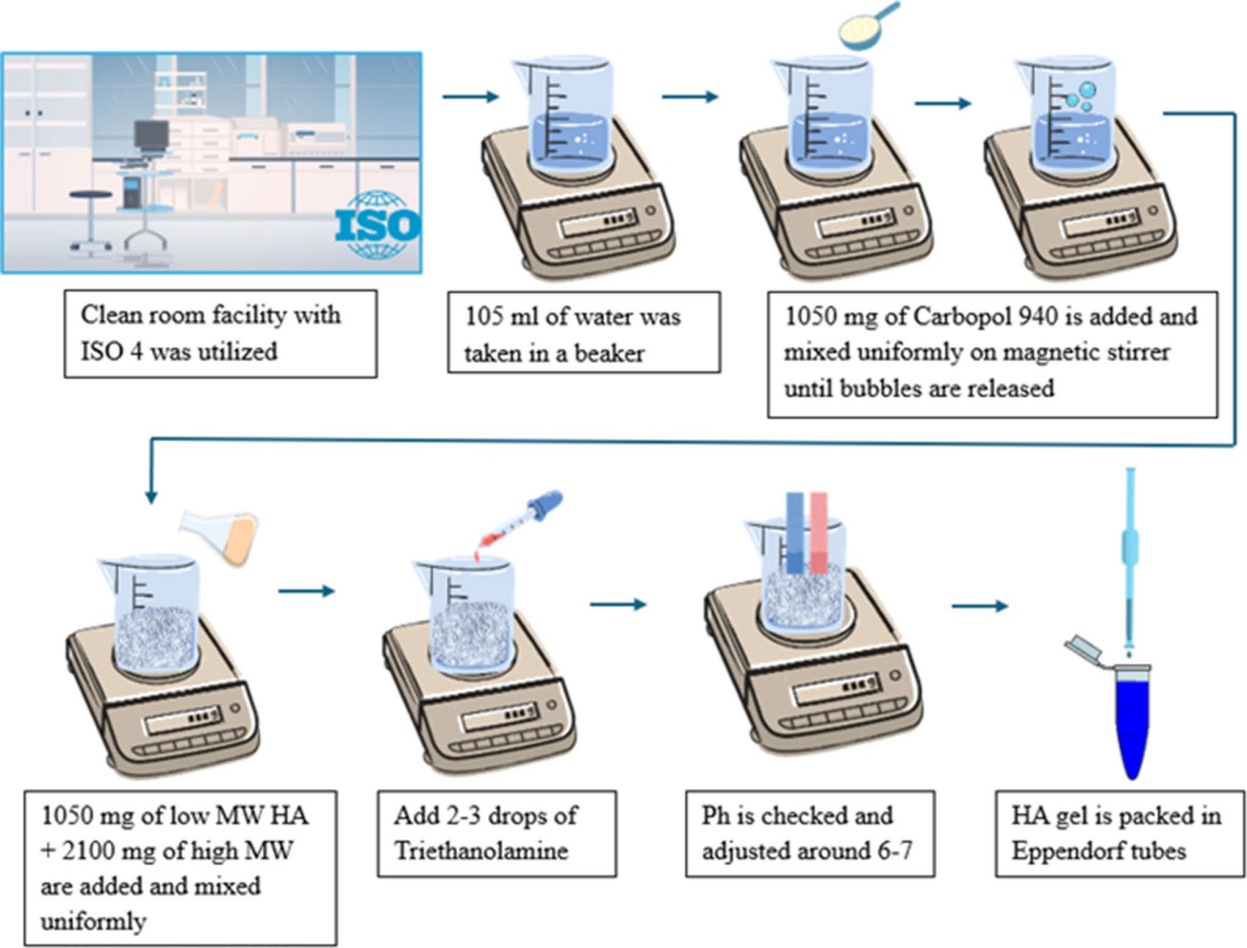
Graphical representation of gel preparation

Group B (HA gel): This group received a custom-formulated HA gel that was prepared specifically for the trial. The concentration of the HA gel was prepared based on the previously published research (Lanka et al. 2022). The gel was prepared in the department of pharmaceutics and in a clean room facility (ISO Class 4). The formulation included 2.1 g of high molecular weight HA and 1.05 g of low molecular weight HA, mixed into 105 mL of distilled water containing 1.05 g of Carbopol 940 (as a gelling agent) and 0.105 g of methylparaben (preservative). The gel was prepared in a controlled clean-room facility (ISO Class 4) to maintain sterility. The preparation process involved continuous stirring of Carbopol in water at 250–300 rpm until the polymer was fully dispersed and de-aerated. The HA powders (low and high molecular weight) were then gradually added and mixed uniformly to ensure even distribution within the gel matrix. Methylparaben was dissolved in the mixture, and finally, 2–3 drops of triethanolamine were added to adjust the pH to about 6.5 (within a range of 6.2–7.0) and to induce gelation by neutralizing the Carbopol. The resulting product was a clear, homogeneous HA gel. The method of HA gel preparation is illustrated in Fig. 5. Both the Blue®m and HA gels were packaged in identical-looking tubes to maintain blinding.

Immediately after suturing, the assigned gel was applied in a thin layer over the surgical site. Patients were instructed to reapply their respective gel three times daily for 7 days using a cotton applicator. No food or drinks were allowed for 30 minutes after each application to ensure adequate contact time of the gel with the wound.

### Clinical outcome measures

Healing and pain outcomes were assessed at designated intervals as follows:

Weeks 1, 2, and 3: Wound healing was evaluated using two indices. The Early Wound Healing Index (EHS, per Lorenzo et al.) assessed three parameters of early healing – closed vs. open incision margins (CSR), presence of fibrin clot (CSH), and tissue redness/inflammation (CSI). In addition, overall healing was graded by the five-point Landry Wound Healing Index, which ranges from very poor (score 1) to excellent (score 5) based on tissue color, response, and granulation characteristics.

Days 1, 2, and 3 (Postoperative): Patient-reported pain was recorded daily using a Numeric Pain Rating Scale (NPRS), an 11-point scale from 0 (no pain) to 10 (worst imaginable pain). Pain ratings were collected via telephone follow-up on each of the first three post-operative days. Telephonic follow up was done by the PI, a standardized pain score scale was used and the response of the patients were noted. No other instructions were provided to the patients.

### Statistical analysis

All analyses were performed using SPSS version 25. Descriptive data were expressed as frequencies and percentages for categorical variables and as median and interquartile range (IQR) for ordinal data. Healing parameters (EHS components and Landry index) and postoperative pain scores (NPRS) were treated as ordinal variables and compared between groups using the Mann–Whitney test. Categorical variables were analyzed using the Chi-square test or Fisher’s exact test where appropriate. A *p*-value < 0.05 was considered statistically significant.

## Results

### Demographics

All 40 participants (20 per group) completed the study. There were no dropouts and no adverse events reported. The mean age of the sample was 37.2 ± 9.1 years. Gender distribution was similar in the two groups. Baseline clinical parameters and surgical sites were comparable between Group A (Blue®m) and Group B (HA), ensuring that any differences in outcomes could be attributed to the interventions.

### Wound healing (EHS and Landry indices)

Both groups exhibited rapid and uneventful healing at the surgical sites. By Week 1, all patients in both groups achieved complete approximation of the incision edges − 100% of surgical sites showed merged incision margins (CSR parameter of EHS) in both Blue®m and HA groups.

In terms of initial fibrin presence along the incision line and tissue redness (CSH parameter of EHS), 20% of sites in each group had a thin fibrin clot at Week 1, while the remaining 80% had no fibrin. By Week 2, fibrin was absent in 100% of sites in both groups, indicating that any early fibrin deposition had completely resolved. Gingival redness and inflammation (CSI parameter of EHS) followed a similar pattern: at Week 1, mild redness was noted in approximately 15–20% of cases in both groups, but not statistically significant (*p* = 0.677). By Week 2, none of the sites in either group exhibited redness, reflecting a return to normal pink color.

As a result of these healing dynamics, the composite Early Wound Healing Score (EHS) which ranges from 0 to 10, was high for both treatments throughout the follow-up. At Week 1, most patients in both groups achieved near-perfect EHS values (scores of 8 to 10 out of 10). By Week 2, all patients in both the Blue®m and HA groups had attained the maximum EHS score of 10, indicating excellent early healing in every case. There was no difference between the groups when the values were assessed using the Mann-Whitney test (*p* > 0.05) (Table 1).

**Table 1:** Comparison of the EHS scores between the groups at different time intervals using the Mann-Whitney test

EHS	Groups	Minimum	Maximum	Median	IQR	p value
Week 1	Blue®m	9	10	10	1.0	0.603
	HA	8	10	10	1.0	
Week 2	Blue®m	10	10	-	-	-
	HA	10	10	-	-	
Week 3	Blue®m	10	10	-	-	-
	HA	10	10	-	-	

**Fig. 6:**
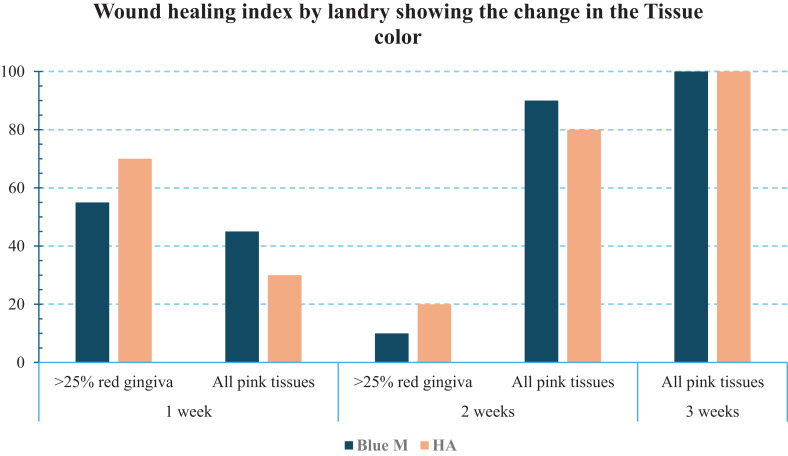
Tissue colour change in subjects based on early wound healing index (EHS) at different time intervals

The longitudinal assessment with the Landry Wound Healing Index likewise demonstrated steady improvement in both groups. At Week 1, the majority of surgical sites in both groups were graded with red/inflamed gingiva (>25% red area), corresponding to lower Landry index scores (indicative of early healing). By Week 3, all sites had transitioned to healthy pink gingival tissues with no significant redness, corresponding to high Landry index scores (reflecting successful wound resolution) in both groups (Fig. 6). In summary, by the end of the third week, clinical healing was deemed excellent for all patients, regardless of whether Blue®m or HA gel was used. No statistically significant differences were observed between the Blue®m and HA groups for any measured healing parameter at any time interval (*p* > 0.05). Chi-square analyses for between-group comparisons of CSR, CSH, CSI, overall EHS scores, and Landry index ratings at each time interval revealed no significant group effect (*p* > 0.05 for all comparisons). The wound healing index - Landry scores between the groups at different time intervals using the Mann-Whitney test showed a *p* value > 0.05 (Table 2).

**Table 2:** Comparison of the wound healing index-Landry scores between the groups at different time intervals using Mann-Whitney test

Time Interval-WHI Landry	Groups	Minimum	Maximum	Median	IQR	p value
Week 1	Blue®m	4.0	5.0	4.0	1.0	0.513
	HA	4.0	5.0	4.0	1.0	
Week 2	Blue®m	4.0	5.0	5.0	0.0	0.382
	HA	4.0	5.0	5.0	0.0	
Week 3	Blue®m	5.0	5.0	-	-	-
	HA	5.0	5.0	-	-	

### Pain scores (NPRS)

**Fig. 7:**
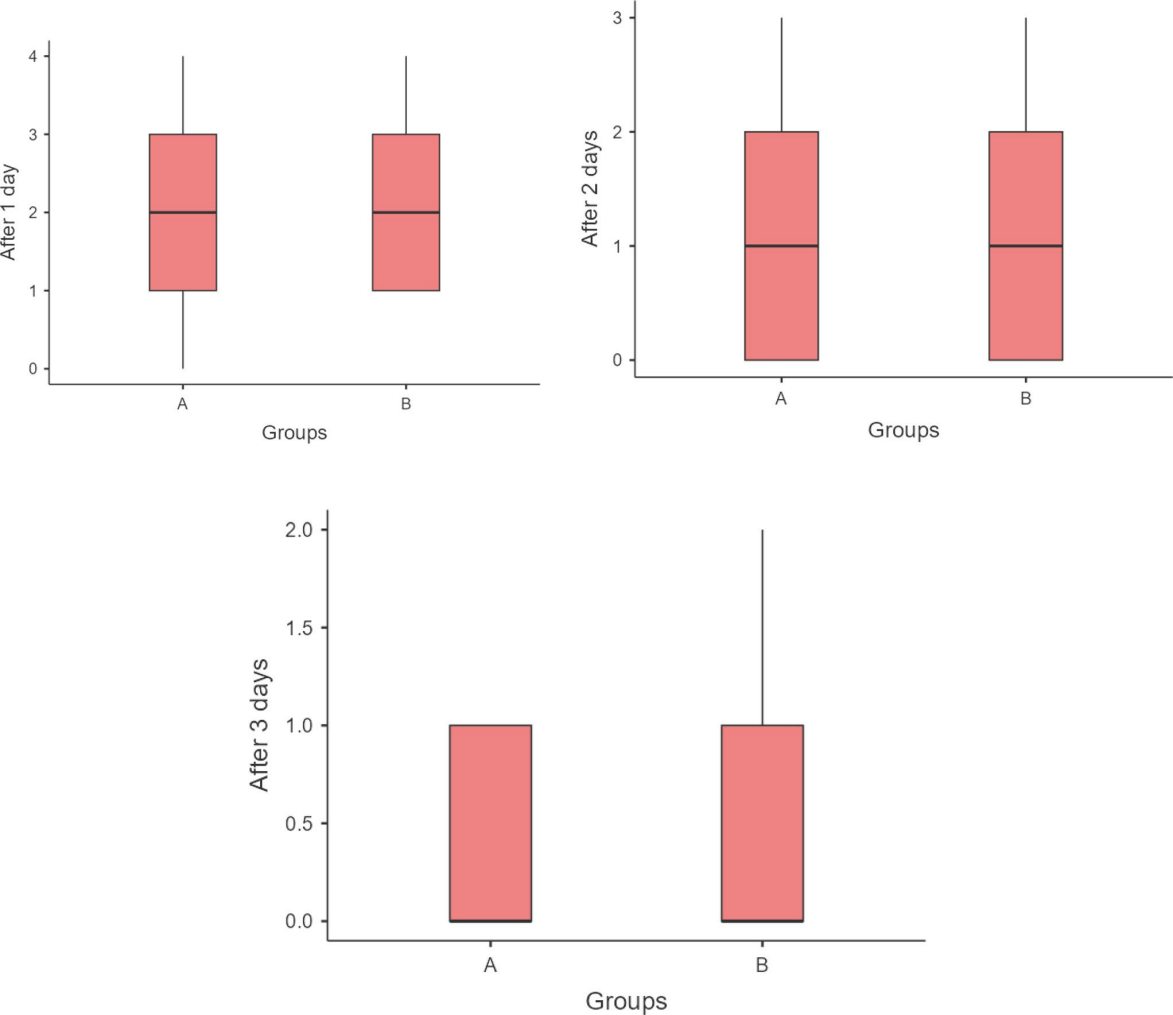
Distribution of the subjects based on wound healing index- Landry at different time intervals

Postoperative pain levels were low in intensity for both groups and diminished rapidly over the first three days after surgery. On Day 1, patients in Group A (Blue®m) reported a mean pain score between 0 and 4, with a median value of 2, whereas Group B (HA) reported scores between 1 and 2, with a median value of 2, both indicating only mild discomfort. By Day 2, average pain ratings had decreased substantially in both groups, and by Day 3, pain was nearly absent. On Day 3, the median rating of NPRS was 1 in both Blue®m group and the HA group. Thus, by 72 hours post-surgery, most patients experienced little to no pain regardless of the gel used. There were no significant differences in pain scores within the groups when the scores were assessed using the chi square test (*p* > 0.05) (Table 3). The pain reduction was very similar for Blue®m and HA, as shown by the similar pain score trends *p* > 0.05 (Mann-Whitney test) (Table 4, Fig. 7). All patients in both groups achieved complete pain relief by the end of the first postoperative week.

**Table 3:** Association of the Numeric pain Rating Scale within the groups

Numeric Pain Rating Scale			Groups		Total	Chi-square value	p value
			Blue®m	HA			
after 1 day	0.0	Count	3	0	3	3.42	0.489
		%	15.0%	0.0%	7.5%		
	1.0	Count	5	7	12		
		%	25.0%	35.0%	30.0%		
	2.0	Count	6	6	12		
		%	30.0%	30.0%	30.0%		
	3.0	Count	5	6	11		
		%	25.0%	30.0%	27.5%		
	4.0	Count	1	1	2		
		%	5.0%	5.0%	5.0%		
after 2 days	0.0	Count	9	7	16	2.64	0.45
		%	45.0%	35.0%	40.0%		
	1.0	Count	5	6	11		
		%	25.0%	30.0%	27.5%		
	2.0	Count	5	3	8		
		%	25.0%	15.0%	20.0%		
	3.0	Count	1	4	5		
		%	5.0%	20.0%	12.5%		
after 3 days	0.0	Count	14	14	28	2.4	0.301
		%	70.0%	70.0%	70.0%		
	1.0	Count	6	4	10		
		%	30.0%	20.0%	25.0%		
	2.0	Count	0	2	2		
		%	0.0%	10.0%	5.0%

**Table 4:** Comparison of the NPRS between the groups at different time intervals using the Mann-Whitney test

NPRS		Minimum	Maximum	Median	IQR	p value
After 1 day	Blue®m	0.0	4.0	2.00	2.0	0.518
	HA	1.0	2.0	2.00	2.0	
After 2 days	Blue®m	0.0	3.0	1.00	2.0	0.426
	HA	0.0	3.0	1.00	2.0	
After 3 days	Blue®m	0.0	1.0	0.00	1.0	0.839
	HA	0.0	2.0	0.00	1.0

Both Blue®m gel and HA gel were associated with low postoperative pain scores, and no statistically significant intergroup differences were observed (*p* > 0.05). No complications or adverse reactions were noted in any patient, and all surgical sites healed satisfactorily. The clinical outcomes suggest that both gels demonstrated comparable performance, with no statistically significant differences detected.

## Discussion

Wound healing following periodontal surgical interventions such as crown lengthening (often involving gingivectomy and flap procedures) is a complex process involving phases of hemostasis, inflammation, proliferation, and tissue remodeling. This process is influenced by the oral environment’s unique challenges, including abundant microbial flora and mechanical stress from oral functions (Wikesj and Nilvéus 1990). The application of topical agents to enhance healing and mitigate postoperative discomfort is a well-established practice in periodontics (Castilla et al. 2012). Both HA and Blue®m gels represent modern, bioactive approaches to postoperative care, moving beyond the passive protection of traditional dressings. Several studies in the literature support the efficacy of both HA and oxygen-based therapies in wound healing. For instance, topical application of HA accelerates palatal wound closure and improves tissue color match, with HA-treated sites achieving complete epithelialization by day 21 compared to day 42 in control sites (no HA) (Eick et al. 2013). This aligns with the current study’s observation that by three weeks (21 days) post-surgery, sites treated with HA gel showed excellent healing. The enhanced early healing with HA can be attributed to its ability to promote re-epithelialization and angiogenesis; HA provides a hydrated matrix that stimulates keratinocyte migration and fibroblast activity, effectively shortening the duration of the inflammatory phase and accelerating the proliferative phase of healing. Consistently, Yildirim’s study also found that postoperative pain decreased more rapidly in patients who received topical HA, with pain scores dropping over a 7-day period in the HA group (Yıldırım et al. 2018). This pain reduction agrees with our findings, suggesting that HA’s anti-inflammatory properties help control postoperative pain as well as enhance tissue repair.

In the present study, both groups showed parallel improvements in the early wound healing indices. By one week, incisions were closed in all cases, and by the third week, complete healing was achieved with both treatments. The Landry index progression in our trial (from predominantly red gingiva at week 1 to entirely pink by Week 3) further underscores the comparable healing trajectory. Kasem et al. (Kasem et al. 2020) observed a similar trend in a study where applying HA after free gingival grafting led to better early healing index scores at days 7 and 14, compared to sites without HA. Our results are in accordance with such findings, reinforcing that HA can positively influence early wound healing outcomes.

Blue®m gel, an oxygen-releasing formulation containing honey enzymes and sodium perborate, is designed to enhance wound repair by elevating oxygen availability in the healing tissues (Leventis et al. 2024). Oxygen plays a critical role in collagen synthesis, angiogenesis, and bacterial clearance, as demonstrated in studies on topical oxygen therapies (Castilla et al. 2012). Juliana et al. found that Blue®m gel, compared to a standard Coe-Pack periodontal dressing after gingival depigmentation surgery, significantly reduced postoperative pain and accelerated re-epithelialization of the gingiva (Kaur et al. 2024). These results are similar to those of our study, and they are attributed to Blue®m’s mechanism of continuously releasing low-dose hydrogen peroxide (active oxygen), which exerts antimicrobial effects and stimulates cell proliferation and vascularization in the wound area. The enhanced oxygenation thus appears to foster a more favourable healing environment and reduce inflammation and pain.

Additional evidence for Blue®m’s efficacy comes from Koul et al., who reported that Blue®m gel was as effective as chlorhexidine gel in reducing periodontal pathogens and inflammation when used as an adjunct to scaling and root planning (Koul et al. 2020). Similarly, Mattei et al. noted that Blue®m mouthwash (with similar active ingredients as the gel) reduced postoperative inflammation and pain in patients undergoing pre-prosthetic surgery (Mattei et al. 2021). These outcomes suggest that Blue®m’s oxygen-releasing mechanism consistently supports early wound stabilization and comfort across different clinical scenarios. This is consistent with the broader evidence that adequate oxygen tension in healing tissues is pivotal for optimal repair, as highlighted by Gordillo and Sen’s work on oxygen in wound healing (Gordillo and Sen 2003).

In contrast, HA gel is a glycosaminoglycan-based biomaterial that promotes wound healing through multiple bioactive properties. HA is highly hygroscopic and mucoadhesive, which helps it maintain a moist wound environment and adhere to the mucosal surface, acting as a barrier and scaffold for cell migration (Gordillo and Sen 2003). It also has immunomodulatory effects, modulating inflammation and facilitating tissue regeneration. These characteristics have been extensively documented in the literature (Casale et al. 2016). In our formulation, we combined both low molecular weight (LMW) and high molecular weight (HMW) HA, an approach supported by in vitro findings. A study by Lanka et al. (2022) demonstrated that a mixture of 5% LMW-HA and 1% HMW-HA yielded the highest viability of human gingival fibroblasts in culture. This provided the rationale for using a blend of HA molecular weights in our gel, aiming to harness the rapid action of LMW-HA (which can stimulate angiogenesis and immune response) alongside the space-filling, anti-inflammatory benefits of HMW-HA.

Multiple clinical studies corroborate the beneficial role of HA in oral wound healing. Eeckhout et al. showed that applying HA (in gel or spray form) after tooth extractions significantly enhanced the rate of wound closure compared to controls (Eeckhout et al. 2022). Lee et al. reported faster healing and reduced pain in patients with recurrent oral ulcers when a 0.2% HA gel was applied, attributing the improvements to HA’s anti-inflammatory and pro-healing properties  (Lee et al. 2008). Jentsch et al. found that an HA spray effectively reduced signs of gingivitis, supporting its use in managing periodontal soft tissue wounds (Jentsch et al. 2003). Additionally, a systematic review by Casale et al. concluded that HA therapies provide significant benefits in healing after oral surgical procedures (Casale et al. 2016). The outcomes of our trial wherein the HA gel treatment led to high healing scores and minimal pain, on par with the Blue®m treatment is in line with these reports and reinforces the notion that HA is a valuable adjunct for periodontal wound management.

The absence of statistically significant differences between Blue®m and HA gels in this study suggests that both agents demonstrated comparable clinical outcomes under the conditions evaluated. Blue®m’s oxygen-driven antimicrobial and proliferative effects likely parallel HA’s hydration-mediated, inflammation-modulating environment in promoting tissue repair (Stern and Jedrzejas 2006). This complementary nature may explain why neither agent outperformed the other under the conditions of our study. Indeed, other investigations have found both oxygen therapy and HA to be effective in various oral wound scenarios (Galli et al. 2008; Schreml et al. 2010).

However, it is important to acknowledge evidence that the efficacy of these agents can be context dependent. For example, Galli et al. conducted a trial where a single application of HA after oral surgery did not significantly improve healing compared to placebo, suggesting that the frequency of HA application or the type of wound may influence its apparent benefit (Galli et al. 2008). Yakout et al. observed that adding a 2% HA gel to photobiomodulation therapy accelerated healing more than photobiomodulation alone, indicating a potential synergistic effect of HA in certain advanced therapies. On the side of oxygen therapy (Yakout et al. 2023), Schreml et al. (Schreml et al. 2010) noted that in well-vascularized wounds with low risk of infection, the benefits of topical oxygen (and by extension, Blue®m gel’s oxygenating action) might be less pronounced, since the baseline oxygen supply is already sufficient. These nuances suggest that while both Blue®m and HA are broadly effective, their relative advantages might emerge in specific clinical situations (e.g., infected vs. non-infected sites, one-time application vs. sustained use, adjunctive therapy combinations, etc.). In our study, the standardized surgical approach (flap crown lengthening in healthy patients) and regular gel applications may have provided an optimal scenario where both treatments could perform at their best, resulting in no statistically significant outcomes.

Pain reduction observed with both gels is supported by prior evidence on their analgesic mechanisms. Blue®m gel’s ability to alleviate pain may stem from improved oxygenation at the wound site, which reduces tissue hypoxia and the associated inflammatory pain response. Sen’s review on oxygen therapy in wound care highlights that adequate oxygen can dampen hypoxia-inducible factors and inflammatory cytokines, thereby lessening pain and swelling (Sen 2009). HA’s analgesic effect is likely due to its capacity to form a protective film over the wound and its intrinsic anti-inflammatory action that soothes the tissue, as noted by Chen and Abatangelo in their overview of HA in wound repair (Leventis et al. 2024). Clinical studies have documented HA’s pain-reducing benefits: Koray et al. reported that topical HA significantly relieved pain in patients with oral mucositis (M et al. 2016), and Nolan et al. observed reduced postoperative pain when HA was applied to periodontal surgical sites (Nolan et al. 2009). Our patients similarly experienced a quick resolution of pain (almost zero pain by Day 3) with both treatments, and the lack of difference between groups suggests that neither agent offers a unique analgesic advantage over the other. Essentially, both interventions provide effective pain control, likely through their respective pathways of reducing inflammation, be it via oxygen delivery or biochemical modulation.

The physical and rheological properties of the gels may also play a role in their performance. HA gel’s viscosity (which depends on the molecular weight composition of HA) contributes to its strong adherence to the gingival surface and a prolonged presence in the wound, potentially extending its therapeutic effects (Brown and Jones 2005). LMW-HA has been shown to enhance immune cell recruitment and angiogenesis (Termeer et al. 2002; West et al. 1985), accelerating the early phases of healing. In contrast, HMW-HA is thought to provide structural scaffolding and sustained anti-inflammatory effects, supporting later stages of repair and tissue integrity. Blue®m gel, by comparison, has a more fluid consistency, which facilitates its spread and penetration into tissue crevices, allowing active oxygen to diffuse uniformly through the wound site (Koul et al. 2020). Each gel also has ancillary benefits: HA is known to have bacteriostatic properties, reducing bacterial adhesion and biofilm formation on wounds, particularly with higher molecular weight formulations, which can protect against infection (Della Sala et al. 2022). Blue®m’s antimicrobial effect through oxygen release similarly combats anaerobic bacteria in the surgical area. It is worth noting that alternative bioactive agents are under investigation as well; for example, Reddy et al. found that the herbal extract gel outperformed HA in healing following gingivectomy in one study (Reddy et al. 2019). Such findings open the door to exploring whether combinations of therapies or new materials might further improve outcomes beyond what HA or Blue®m alone can achieve.

Overall, the findings of this study indicate that Blue®m gel and HA gel are both safe and effective adjuncts for enhancing soft tissue healing and controlling postoperative pain following functional crown lengthening surgery. This suggests that clinicians can confidently use either Blue®m or HA gel as a topical application agent after crown lengthening procedures. The selection of one over the other may therefore be guided by practical factors such as product availability, cost considerations, or personal preference of the clinician or patient. By leveraging such bioactive wound dressings, periodontal surgeons can improve patient comfort and possibly expedite recovery, thereby adding a valuable dimension to post-surgical care beyond traditional protective dressings.

## Conclusion

Both Blue®m gel and hyaluronic acid gel significantly promote soft tissue healing and provide postoperative pain relief following functional crown lengthening. There were no statistically significant differences in healing or pain parameters between the groups. Both Blue®m gel and hyaluronic acid gel demonstrated favorable clinical outcomes in soft tissue healing and postoperative pain control following functional crown lengthening. No statistically significant differences were observed between the groups for any measured parameter.

## Limitations

The study was a single-centered clinical research.

Although the study was patient and assessor-blinded, the principal investigator performed both the surgical procedures and postoperative pain follow-up, which may introduce potential performance and detection bias.

Short follow-up period (3 weeks) to assess the healing objectively, but a more comprehensive and histological assessment could be done with various biomarkers of inflammation.

Pain was evaluated only through subjective self-reports (NPRS).

No microbiological parameters were analysed to corroborate clinical findings.

## Electronic supplementary material

Below is the link to the electronic supplementary material.

Supplementary material 1

## Data Availability

Data to be made available on request.
