# Supplementary material for: Blue®m gel vs hyaluronic acid gel in wound healing and pain control following functional crown lengthening: a randomized controlled trial
Source: Saudi Dent J. 2026 May 12;38(5):65. doi: 10.1007/s44445-026-00178-4 (PMC13168397; doi:10.1007/s44445-026-00178-4)
Supplement: Supplementary file 1 — Supplementary material 1 [file 44445_2026_178_MOESM1_ESM.docx]

**Table: Association of Early Wound Healing Index with the Groups**

| **EWHI** | **Time Interval** | **Score** | **Blue^®^m** | **HA** | **Total** | **Chi-square** | **p value** |
| --- | --- | --- | --- | --- | --- | --- | --- |
| **CSR** | Week 1 (T1) | 6 | 20 (100%) | 20 (100%) | 40 (100%) | – | – |
| **CSH** | Week 1 | 1 | 4 (20.0%) | 4 (20.0%) | 8 (20.0%) | 0 | 1 |
|  |  | 2 | 16 (80.0%) | 16 (80.0%) | 32 (80.0%) |  |  |
| **CSI** | Week 1 | 1 | 4 (20.0%) | 3 (15.0%) | 7 (17.5%) | 0.173 | 0.677 |
|  |  | 2 | 16 (80.0%) | 17 (85.0%) | 33 (82.5%) |  |  |

*Statistical comparison not performed where all observations were identical across groups*.

**Table: Distribution of the subjects based on Early Wound Healing Index (CSI) at different time intervals**

| **Parameter** | **Time** | **Category / Score** | **Blue^®^m** | **HA** | **Total** | **χ²** | **p value** |
| --- | --- | --- | --- | --- | --- | --- | --- |
| **Score** | Week 1 | 4 | 12 (60.0) | 14 (70.0) | 26 (65.0) | 0.44 | 0.507 |
|  |  | 5 | 8 (40.0) | 6 (30.0) | 14 (35.0) |  |  |
|  | Week 2 | 4 | 2 (10.0) | 4 (20.0) | 6 (15.0) | 0.784 | 0.376 |
|  |  | 5 | 18 (90.0) | 16 (80.0) | 34 (85.0) |  |  |
|  | Week 3 | 5 | 20 (100.0) | 20 (100.0) | 40 (100.0) | – | – |
| **Tissue colour** | Week 1 | <25% red gingiva | 11 (55.0) | 14 (70.0) | 25 (62.5) | 0.96 | 0.327 |
|  |  | All pink tissues | 9 (45.0) | 6 (30.0) | 15 (37.5) |  |  |
|  | Week 2 | <25% red gingiva | 2 (10.0) | 4 (20.0) | 6 (15.0) | 0.784 | 0.376 |
|  |  | All pink tissues | 18 (90.0) | 16 (80.0) | 34 (85.0) |  |  |
|  | Week 3 | All pink tissues | 20 (100.0) | 20 (100.0) | 40 (100.0) | – | – |

*Bleeding on probing, granulation tissue, exposed connective tissue at the incision margin, and suppuration were absent in all subjects at Weeks 1, 2, and 3 in both groups; therefore, no statistical comparison was performed for these parameters due to lack of variability.*
